# School policies, built environment and practices for non-communicable disease (NCD) prevention and control in schools of Delhi, India

**DOI:** 10.1371/journal.pone.0215365

**Published:** 2019-04-18

**Authors:** Shalini Bassi, Vinay K. Gupta, MinHae Park, Gaurang P. Nazar, Tina Rawal, Soumyadeep Bhaumik, Kanwal Preet Kochhar, Monika Arora

**Affiliations:** 1 Health Promotion Division, Public Health Foundation of India (PHFI), Gurgaon, Haryana, India; 2 Department of Health Services Research and Policy, Faculty of Public Health and Policy, London School of Hygiene and Tropical Medicine (LSHTM), London, United Kingdom; 3 Department of Physiology, All India Institute of Medical Sciences (AIIMS), Ansari Nagar, New Delhi, India; Griffith University, AUSTRALIA

## Abstract

**Objective:**

To assess school policies, built environment and practices for prevention and control of non-communicable diseases in schools of Delhi, India.

**Methods:**

School built environments and policies were assessed using a structured observation checklist in 10 private and 9 government schools which were randomly selected from all 184 co-educational schools with primary to senior secondary level education in Delhi, India. A self-administered questionnaire was also completed by teachers from each school (n = 19) to capture information specific to school policies. Surveys were also conducted with parent of students in class II (aged 6–7 years; n = 574) and student in class XI (aged 15–16 years, n = 755) to understand school practices.

**Results:**

The majority of government (88.9%; n = 8) and private (80%; n = 8) schools reported having comprehensive school health policy. In terms of specific health behaviours, policies related to diet and nutrition in government schools were mostly restricted to primary levels with provision of the mid-day meal programme. All schools had two physical education periods per week of about 45–50 minutes. Most schools were compliant with tobacco-free school guidelines (n = 15 out of 19) and had alcohol control policies (n = 13 out of 19). Parent and student reports of practices indicated that school policies were not consistently implemented.

**Conclusion:**

Most schools in Delhi have policies that address health behaviours in students, but there was considerable variation in the types and number of policies and school environments. Government schools are more likely to have policies in place than private schools. Further work is needed to evaluate how these policies are implemented and to assess their impact on health outcomes.

## Introduction

Rapid changes in India’s demography, socio-economic profile, and lifestyles have led to the emergence of non-communicable diseases (NCDs) as a leading cause of morbidity and mortality. Currently, NCDs account for over three-quarters of deaths in adults aged over 50 years, and almost half of all deaths among younger adults [1].

The onset of NCDs can be prevented or delayed by addressing key modifiable risk factors, including unhealthy diet, physical inactivity, tobacco use and alcohol abuse [2,3]. These lifestyle behaviours are often formed early in life, and track into adulthood [4–7]. Interventions to promote healthier lifestyles are potentially more effective when implemented pre-adulthood, before unhealthy lifestyles become established [8–10].

Schools have been widely recognized as an important platform for delivering health promotion interventions [11,12]. Despite recognition of their important role, little is known about existing initiatives and approaches to behaviour that are currently being implemented in the Indian context. The objective of this study was to assess existing school policies, built environment and practices regarding prevention and control of NCDs in private and government schools in Delhi, India.

## Methods

### Sample

A cross-sectional study was conducted between October 2013 and September 2015 in 19 randomly selected schools (10 private and 9 governments), sampled from all co-educational schools with primary (I-V class) to senior-secondary level (XI-XII class) in New Delhi, India. These 19 schools covered approximately 10% of the 184 schools of the state (84 government and 100 private schools) [13]. We received permission from school authorities, written informed consent from parents of students and written informed assent from students.

### School policies

School policies in relation to four major modifiable NCD risk factors: unhealthy diet, physical inactivity, tobacco and alcohol use, were studied. Policies were defined within the realms of both written guidelines (*formal written guidelines being implemented by the school administration)* and unwritten norms (*which are being implemented at school level either through spoken instructions or vide norms*).

Policies were selected based on the findings of a recent systematic review of school policies for NCD risk factor reduction [11] and the Comprehensive School Health Guidelines of Central Board of Secondary Education (CBSE) under the Comprehensive School Health Programme (CSHP). The CSHP focuses on developing healthy attitudes and enhancing life-skills of students to overcome the multiple health concerns affecting young people. This programme is integrated within the educational system at the national and state levels to facilitate the holistic approach to child and adolescent development in schools. The programme also utilises available educational opportunities for health promotion including formal, informal and innovative approaches in curricular pedagogy [14]. The selected policies were categorised as follows: food and nutrition (*number of nutrition education periods and activities*, *nutrition curriculum*, *access to food outlets around the school campus during school hours*); canteen policy (*nutritional guidelines for foods available at canteen*, *hygiene monitoring*, *availability of healthy food options*, *no sale of fried/carbonated/sweetened beverages/salty foods*, *regular monitoring and revision of canteen menu*, *pricing guidelines to encourage sale of healthy foods*); lunch box policy (*written guidelines for sending healthy food in lunch boxes*, *regulation of foods brought from home*, *regular monitoring of lunch box*, *fruit breaks*); sports/physical activity (*promote physical activity through intra/inter school competitions*, *availability of adequate and well maintained play grounds*, *effective utilization of Physical Education period by ensuring every child is physically active and engaged*, *availability of sports equipment*); tobacco and alcohol control (*information about harmful use of tobacco and alcohol in the school curriculum*, *no sale of tobacco and alcohol inside or within/around the school campus; display of mandatory boards)*.

### Data collection

Data were collected using three methods: teacher questionnaire, observation checklist, parent and student survey. A self-administered questionnaire was completed by the principal/teacher from each school (n = 19) to capture information regarding the seven policies outlined in the CSHP of the CBSE [14] and any other specific school policies related to the four modifiable risk factors for NCDs.

School built environment (appropriate facilities, infrastructure and surrounding environment) and policies were assessed through a structured observation checklist. The checklist was developed following a scoping review of school environment assessment tools [15, 16]. Observations were completed by trained research staff over 2–3 schooldays, including physical education periods and lunch breaks. All observations were completed during school hours and the research staff completed these observations independently, but with prior permission from the school authorities. Observers were trained in passive observation to ensure their presence had minimal impact on school activities.

The quality of playground and sports equipment were rated as ‘Good’ (*clearly fit for its purpose*), ‘Adequate’ (*show some damage or maintenance failure*, *but does not impair use*) and ‘Poor’ (*damaged to the extent that it is no longer useable*, *or fit for purpose*). The ‘usage of school ground’ was assessed through criteria: ‘very suitable’ (*if they have different areas where children can play during the lunch break*), ‘somewhat suitable’ (*if there is a single restricted space for children to play in*), and ‘not suitable’ (*if play space is very limited or absent*)’.

To understand school practices, surveys were conducted with parents of children studying in class II (aged 6–7 years; n = 574) and students in class XI (aged 15–16 years, n = 755). From each school, one section was randomly selected from classes II and XI, and formed the sample for the parents and students survey. The two age groups were selected to reflect differences in school policies and practices for young children and late adolescents. At age 6–7 years, children’s behaviours will be influenced more strongly by parents, teachers and the immediate school environment, while in late adolescence students may have greater independence and flexibility during school hours. All study tools were piloted in one government and one private school to ensure contextual relevance, feasibility and reliability, data for which is not reported in the manuscript.

### Ethical clearance

Permissions for the study were obtained from the Directorate of Education (DoE) and the School Health Scheme, Government of NCT of Delhi. Ethical approval was obtained from the Public Health Foundation of India’s Institutional Ethics Committee (*TRC-IEC-204/13*) and Research Ethics Committee at London School of Hygiene and Tropical Medicine (*7368*).

## Data analysis

All responses from the observations and questionnaires were summarized as frequencies and percentages using statistical software Stata v.12.0 (Stata Corp, Texas). We described the types of policies reported by teachers, out of the seven listed policies. Results are presented by type of school (private or government) and grade (primary and senior secondary). Results from the parent and student survey were also stratified according to the presence (or otherwise) of school policies as reported by teachers or observations.

## Results

The 19 schools (10 private and 9 government) that participated in the study were located in 11 of the 13 districts in Delhi. The overall sample (n = 1329) consisted of 59% male students, 46% students from government schools and 44% students from grade two. The student to teacher ratio was 29 with an average of 36 students per class in private and 34 students per class in government schools. A total of 342 parents of class II students (response rate 84%) and 371 students in class XI (response rate 87%) in government schools, and 232 parents of class II students (response rate 76%) and 384 class XI students (response rate 91%) in private schools took part in the study. All teachers approached (n = 19) participated in the survey.

Table 1 summarizes school health policies as reported by teachers. Overall, 80% (n = 8) of private and 88.9% (n = 8) of government schools reported having CBSE’s CSHP. All government schools provided a free and balanced lunch to all students of primary and upper primary classes under the government’s mid-day meal scheme [17]. Most private schools had physical activity (n = 9, 90%) and lunchbox policies (n = 7, 70%), but tobacco and alcohol control policies were only reported in around half of the schools.

**Table 1:** School health policies by class and type of school reported by teachers.

	Private schools (n = 10)		Government schools (n = 9)	
School Policies	Primary (class 1–5)	Senior secondary (class 11–12)	Primary (class 1–5)	Senior secondary (class 11–12)
	N(%)	N(%)	N(%)	N(%)
Comprehensive School Health Policy (CSHP)	7 (70)	8 (80)	8 (88.9)	8 (88.9)
Food/nutrition policy	6 (60)	6 (60)	8 (88.9)	6 (66.7)
Canteen Policy	5 (50)	5 (50)	N.A^a^	N.A^a^
Lunch box policy	7 (70)	6 (60)	N.A^b^	4 (44.4)
Sports / physical activity policy	9 (90)	9(90)	8 (88.9)	8 (88.9)
Tobacco control policy (Tobacco-free school guidelines)	7 (70)	6 (60)	8 (88.9)	8 (88.9)
Alcohol control policy	5 (50)	5 (50)	8 (88.9)	7 (77.8)

^a^N.A = Not Available, none of the government schools had a canteen;

^b^Mid-day meal is provided to students of primary (classes 1-V) and upper primary classes (VI-VIII) in government schools so children usually do not bring lunch from home

Table 2 summarizes school health policy and practices reported by teachers. Ninety percent of private schools (n = 9) reported monitoring of lunch boxes for primary classes during recess. On an average, there were two physical education periods per week with median duration of 50 minutes in government and 40 minutes in private schools. It was reported that a physical education curriculum was present for almost all primary (n = 8, 80%) and senior secondary (n = 9, 90%) classes in private schools, and in 77.8% (n = 7) in primary and all senior secondary classes in government schools.

**Table 2:** School health policies and practices as reported by teachers (n = 19).

	Private schools (n = 10)		Government schools (n = 9)	
Policies/Practices	Primary (class 1–5)	Senior secondary (class 11–12)	Primary (class 1–5)	Senior secondary (class 11–12)
	N(%)	N(%)	N(%)	N(%)
**A) Policies and practices related to nutrition /diet**				
Nutrition curriculum	4 (40)	4 (40)	3 (33.3)	4 (44.4)
Specifies number of nutrition education periods and activities at school	2 (20)	1 (10)	3 (33.3)	4 (44.4)
Regular training of school staff on diet/nutrition related issues	3 (30)	3 (30)	6 (66.7)	6 (66.7)
Nutrition education extends beyond the school environment to parents and community	7 (70)	5 (50)	8 (88.9)	8 (88.9)
Nutritional guidelines for foods available at canteen/ school meal	5 (50)	5 (50)	0 (0.0)	0 (0.0)
Monitoring of hygiene in school meal/canteen	7 (70)	7 (70)	4 (44.4)	N.A^a^
Sale of fried foods in canteen is prohibited	4 (40)	4(40)	N.A^a^	N.A^a^
No sale of carbonated/sweetened beverages in canteen	6 (60)	6(60)	N.A^a^	N.A^a^
No sale of salty foods e.g. wafers, chips in canteen	3 (30)	4 (40)	N.A^a^	N.A^a^
Pricing guidelines for canteens to encourage sale of healthy food choices at reduced cost	7(70)	6(60)	N.A^a^	N.A^a^
Regular monitoring of lunch boxes at school	9 (90)	5 (50)	0(0.0)	4 (44.4)
Regulations regarding the sale of food outside the school by vendors	6 (60)	5 (50)	1 (11.1)	1 (11.1)
**B) Policies/practices related to physical activity**				
Physical education curriculum	8 (80)	9 (90)	7 (77.8)	9 (100)
PE marks/grades have weightage in final examination	9 (90)	8 (80)	2 (22.2)	8 (88.9)
Inclusion of yoga in school	7 (70)	6 (60)	6 (66.7)	7 (77.8)
Promotes physical activity through intra and inter school competitions	10 (100)	9 (90)	9 (100)	9 (100)
Community use of school facilities for physical activity outside of the school day	5 (50)	5 (50)	4 (44.4)	4 (44.4)
**C) Policies/norms/provisions about tobacco and alcohol in schools**				
School curriculum includes information about harmful effects of tobacco	4 (40)	6 (60)	7 (77.8)	8 (88.9)
School curriculum includes information about harmful effects of alcohol	4 (40)	7 (70)	7 (77.8)	8 (88.9)
School has provision to sensitize parents/visitors/ non-teaching staff about harmful effects of tobacco use	6 (60)	7 (70)	8 (88.9)	8 (88.9)
School has provision to sensitize parents/visitors/non-teaching staff about harmful effects of alcohol use	5 (50)	6 (60)	8 (88.9)	8 (88.9)
School prohibits use and sale of tobacco inside the school campus	9 (90)	8 (80)	9 (100)	9 (100)
School prohibits sale of tobacco within 100 yards of school campus	8 (80)	7 (70)	9 (100)	9 (100)
School has policies/rules to prohibit smoking/tobacco use within the school campus	9 (90)	8 (80)	9 (100)	9 (100)
School has policies/rules which prohibit sale of alcohol around the school campus	8 (80)	7 (70)	9 (100)	9 (100)

^a^N.A = Not Available, none of the government schools had a canteen, so information pertaining to canteen related policies is missing

Most government schools (n = 7; 77.8% at primary and n = 8; 88.9% at senior secondary) reported having information about harmful effects of tobacco and alcohol in the school curriculum. Private schools had similar information in their curriculum in only 40% (n = 4) of primary and 60% (n = 6) of senior secondary classes. Teachers from all government and 90% (n = 9) of private schools reported prohibition of sale and use of tobacco inside the school campus. All government and 80% (n = 8) of private schools prohibited the sale of tobacco products within 100 yards of school campus.

Table 3 summarises the findings of the school built environment assessment via structured observations. While all schools had a playground, the playgrounds were suitable for sports in 60% of the private schools (n = 6) and 33.3% (n = 3) of the government schools. Adequate or good sports equipment were available in all private and 71.4% (n = 5 out of 7) of government schools.

**Table 3:** Assessment of school built environment in relation to NCD risk factors through structured observations.

	Private schools (n = 10)	Government schools (n = 9)
	N (%)	N (%)
**1) Policies on physical activity**		
**Play ground in schools**	10 (100)	9 (100)
**Number of playground equipment items**		
0	4 (40)	2 (22.2)
1–5	2 (20)	5 (55.6)
More than 5	4 (40)	2 (22.2)
**Quality of playground equipment** (n = 6 Private, 7 Government)†		
Good	2 (33.3)	1 (14.3)
Adequate	4 (66.7)	4 (57.1)
Poor	0 (0)	2 (28.6)
**School grounds are suitable for**		
**Sports**		
Very suitable	6 (60)	3 (33.3)
Somewhat suitable	2 (20)	2 (22.2)
Not suitable	2 (20)	4 (44.4)
**Informal games**		
Very suitable	5 (50)	4 (44.4)
Somewhat suitable	3 (30)	2 (22.2)
Not suitable	2 (20)	3 (33.3)
**Other outdoor games**		
Very suitable	4 (40)	3 (33.3)
Somewhat suitable	3 (30)	2 (22.2)
Not suitable	3 (30)	4 (44.4)
Pedestrian/ walking area near the school	2 (20)	1 (11.1)
**2) Policies on nutrition**		
School has canteen	6 (60)	0 (0)
Food vendors/hawkers selling high fat, high salt and high sugar drinks within 500 yards of schools campus	0 (0)	0 (0)
Students getting food from food outlets or venders during school hours	0 (0)	0 (0)
**Signage in /outside the school campus**		
a. Food sponsoring company	1 (10)	0 (0)
b. Beverage sponsoring company	3 (30)	0 (0)
c. School nutrition policy statement	1 (10)	5 (55.6)
d. School physical activity policy statement	1 (10)	1 (11.1)
e. School health policy statement	1 (10)	1 (11.1)
**3) Tobacco Control Policies**		
Tobacco vendors within a radius of 100 yards from the school campus	4 (40)	1 (11.1)
Smoking in the school campus	0 (0)	0 (0)
Tobacco litters/tobacco related products (cigarettes/bidi buds, smokeless tobacco wrappers, lighters, ashtrays etc.) in the school	2 (20)	0 (0)
Declared as “No smoking area”	1 (10)	8 (88.9)
Sale of tobacco products is banned	2 (20)	7 (77.8)
**4) Alcohol control policies**		
Alcohol outlet in the immediate vicinity of the school	1 (10)	0 (0)

^†^ The number of schools represents schools having playground equipments

None of the government schools had a canteen, while 60% of private schools had one. None of the schools had food vendors/hawkers selling food high in fat and salt and/or carbonated drinks within 500 yards of the school campus. Signage of food and beverage companies (in and outside school campus) were observed in private schools (10% and 30% respectively), but not in any government schools. One private (10%) and five government (55.6%) schools displayed signs related to school nutrition policy.

Tobacco vendors within a 100-yards (*91*.*44 meters*) radius of the school were observed in 40% (n = 4) of private and 11.1% (n = 1) of government schools. Most government schools had “*No smoking Area-Smoking here is an offence*” (n = 8; 88.9%) and “*Sale of Tobacco Products is banned*” boards (n = 7; 77.8%) on display, whereas only one private school had such displays. One private school had an alcohol outlet in its immediate vicinity.

Table 4 summarises parent and student reports of school policies, practices and environment, according to the school policies reported by teachers. Almost half of parents (48.6%) of primary students at private schools that did not have a canteen, reported that their child’s school had a canteen. Approximately 21.5% of parents from private schools reported vendor access during school hours which reported that such access was prohibited. Almost 79.8% of students reported marking of physical education classes where physical education policy existed. In schools with tobacco control policies, 47.3% parents and 51.1% students reported display of “*Tobacco Free School*” sign boards. Similarly, sale of tobacco and alcohol within/around school campuses was reported by a lower proportion of students and parents where these policies existed as compared to where policies were lacking.

**Table 4:** Practices and environment in relation to major NCD risk factors as reported by students of Grade 11, parents of Grade 2 students by existence of policy in school (as reported by teachers).

Policy reported by teachers	Presence of policy reported by Parent and Student N (%)†	
	Parents	Students
**Availability of canteen in the school (only in Private schools)**		
No (n* = 5)	84 (48.6)	56(34.4)
Yes (n = 5)	141 (84.4)	171(86.4)
**Restriction on the sale of foods high in fat, salt and sugar in school’s canteen**		
No (n = 5)	13(7.8)	23(15.1)
Yes (n = 5)	25(15.6)	70(35.2)
**Accessibility to vendors around school campus during school hours**		
No (n = 5)	31 (21.5)	28 (14.4)
Yes (n = 14)	133 (30.9)	76 (13.6)
**Marking for Physical Education classes in school**		
No (n = 2)	23 (31.9)	43 (74.1)
Yes (n = 17)	118 (23.5)	541 (79.8)
**Accessibility to playground during school hours**		
No (n = 2)	10 (13.9)	40 (46.5)
Yes (n = 17)	86 (17.1)	392 (58.6)
**Accessibility to sports/ playing equipment during school hours**		
No (n = 2)	4 (5.6)	16 (18.6)
Yes (n = 17)	34 (6.8)	202 (30.2)
**Signage of “Tobacco Free School” at a prominent place near entrance**		
No (n = 4)	49(36.8)	23(22.3)
Yes (n = 15)	196(47.3)	328(51.1)
**Signage of “No smoking- smoking here is an offence” in school campus**		
No (n = 4)	70(52.6)	36(35.3)
Yes (n = 15)	241(58.4)	407(64.0)
**Sale of tobacco within 100 yards of school campus**		
No (n = 4)	53(39.6)	46(44.2)
Yes (n = 15)	97(23.4)	213(33.3)
**Sale of alcohol within 75 meters of school campus**		
No (n = 6)	48(23.9)	40(23.1)
Yes (n = 13)	36(10.3)	68(11.8)

*n represents combined number of private and government schools with specific policy in primary classes as reported by teachers

^†^ Percentages are presented only for the parents and students who reported the specific practice.

## Discussion

To the best of our knowledge, this is the first study to comprehensively describe school policies, built environment and practices in relation to NCD risk factors in the state of Delhi, India. Almost all participating schools in Delhi had some policies and practices in place to address NCD risk factors. However, there was considerable variation in the types and number of policies in place and in school environments. There were discrepancies between school practices as reported by parents and students and those reported by teachers, and as per observations, suggesting gaps between stated policies and perceived or actual implementation.

Most schools regulated access to unhealthy foods, had nutritional guidelines, monitored canteen menus, and did not allow sale of carbonated/sweetened beverages and food high in fat and/or salt within the school’s campus. In general, such policies were more common in government schools than in private schools. It is known that availability of unhealthy foods in school influences children’s food choices and behaviours [18, 19]. However, policies related to diet and nutrition were more frequently reported at primary than senior secondary levels, even though it is known that the transition phase in late adolescence has an important influence on healthy eating behaviours [20]. Most private schools had policies to prevent the sale of food products near school campuses but government schools did not. In 2013, the Delhi High Court directed the Delhi government to ban junk food and carbonated drinks in schools, its sale within 500 yards of the institutions and junk food advertisements targeting children [21]. In 2015, the Food Safety Standards Authority of India (FSSAI) in its draft guidelines had recommended restricting and limiting the availability of foods high in fat, sugar and salt in schools and in the area within 50 meters of school [22]. In government schools, adoption and dissemination of these guidelines is expected to drive change in school-level policies. Even in schools which reported such policies, parents and students reported the availability of food vendors around the school. As such, implementation of policies beyond the school remain a challenge.

All the schools had playgrounds and physical education periods for each class. There were however only two PE periods per week with median duration of 40–50 minutes, which is less than the minimum duration in the CBSE guidelines which recommends “40–45 minutes of physical activities/games for classes I to X every day and all the students from classes XI and XII to participate in physical activity at least twice a week” [23].

In terms of tobacco related school policies and practices, compliance with Section 6 of the Indian Tobacco Control Act 2003, i.e. sale of any kind of tobacco products is banned within the radius of 100 yards of any educational institute, was observed in all government schools [24]. Most schools were also compliant with the compulsory guidelines issued by the CBSE to make schools tobacco-free [25]. The majority of the government and private schools have alcohol related polices and education about harmful effects of alcohol use in the school curriculum. However, both tobacco and alcohol-related policies were reported less frequently for private schools than government schools, indicating that these are areas which may require more robust regulation and enforcement.

A key strength of the current study is the ability to triangulate information from multiple sources through diverse data collection approaches and the holistic nature of assessment wherein policies, built environment as well as practices were assessed. The study thereby enables us to provide a more comprehensive picture of the policies and practices beyond just what is officially documented. However, the findings of our study may not be generalizable to schools that are not governed by the DoE and other schools in India. We also acknowledge that the high proportion of reported adoption of policies might be a result of the fact that only schools approved by the DoE were included. There is a need for future studies involving all schools irrespective of the affiliation and approval status. It is also important to conduct an equity-focused study to understand how the school policies affects different categories of students. There is also need for further studies in rural areas of the country, where adoption of policies as well as their implementation might be more challenging.

## Conclusion

While India makes an epidemiological transition with NCDs adding to the disease burden there is a need to mount an education sector response for prevention and control of NCDs. While a range of policies and practices are in place, there is a need for filling gaps, developing synergies across sectors and effective implementation. An evidence-based education sector response to NCD risk factors targeting the critical transition periods of early life, including adolescence, is required [26, 27]. While schools have a central role in influencing students’ behaviours, an effective response will require participation from families and cross-sector collaboration with providers of social and community services, including youth clubs and sports centres, which provide further opportunities for shaping young people’s behaviours.

## Supporting information

S1 File Study tools. (PDF)

S2 File Data sets(de-identifier). (ZIP)
