# Supplementary material for: School policies, built environment and practices for non-communicable disease (NCD) prevention and control in schools of Delhi, India
Source: PLoS One. 2019 Apr 18;14(4):e0215365. doi: 10.1371/journal.pone.0215365 (PMC6472740; doi:10.1371/journal.pone.0215365)
Supplement: S1 File — (PDF) [file pone.0215365.s001.pdf]

School ID (For Office Use Only)________

School Policy Assessment
Teacher Questionnaire

Instructions to fill the questionnaire:

- This questionnaire you are about to complete is important. The information you share with us will be used to develop health and nutrition programmes for children and youth.
- Please answer all the questions. Kindly note that there is no correct or wrong answer
- Choose the most appropriate answer according to you.
- Please be as honest as you can with all of your responses.
- For every question or its sub-parts, choose only one answer (unless specified).
- Some questions are open-ended and have blank spaces provided in front of them. Use these spaces to provide necessary information
- This questionnaire may take you around 15-20 minutes to complete.

A. School Demographics

1. Name of the school: ______________________

2. Your designation at school _________________

3. Is your school: Government school ☐ Private school ☐

4. What is the total number of students in your school? ______students

5. What is the total number of teachers in your school? ______teachers

6. How many Physical Education teachers/ coaches are there in your school?
   ________ teachers/ coaches

7. How many Yoga teachers are there in your school? ______teachers

8. Does your school time table have regular Physical Education/Games (including Yoga and PT periods) at school (please specify number of periods /week)?

	No	Yes	Number of periods/ week	Duration (in minutes)
a. Primary (Class I – V)				
b. Middle (Class VI – VIII)				
c. Higher secondary (Class IX – X)				
d. Senior secondary (Class XI – XII)				

9. Does your school time table have regular Nutrition Education periods at school (please specify number of periods /week)?

	No	Yes	Nutrition education is integrated in other subjects/curriculum e.g. science	Nutrition Education is an optional subject
a. Primary (Class I – V)				
b. Middle (Class VI – VIII)				

c. Higher secondary (Class IX – X)				
d. Senior secondary (Class XI – XII)				

10. Does your school have a school health programme?
   a. Yes ☐
   b. No ☐

10a. If yes,
   a. CBSE comprehensive school health programme ☐
      (e. g. school health club, school health manuals etc.)
   b. Any other programme (please specify) __________________ ☐

10 b. Please specify the classes for which the programme is applicable _____________ ____________

11. Does your school have health advisory committee or school health committee?
   a. Yes ☐
   b. No ☐

12. Does your school have the following health policy/ies? (Mark all that apply)

**Policy**	**Written**				**Unwritten**				**No policy**	**Not Applicable**
	**Primary**	**Middle**	**Higher secondary**	**Senior secondary**	**Primary**	**Middle**	**Higher secondary**	**Senior secondary**		
Food/nutrition policy										
Canteen Policy										
Lunch box policy										
Sports / physical activity policy										
Tobacco control policy										
Alcohol control policy										
Comprehensive School Health Policy										

13. Who among the following are engaged in formulating health policies at your school? (Mark all that apply)
    a. Director/Principal ☐
    b. Board members ☐
    c. School health committee ☐
    d. Teaching staff ☐
    e. Parents ☐
    f. Others (please specify) ________________________

B. Nutrition Education at School

14. Do your school level policies/rules/regulations cover the following aspects of nutrition education?

	Yes				No	Not Applicable
	**Primary**	**Middle**	**Higher secondary**	**Senior secondary**		
a. Nutrition curriculum						
b. Specifies number of nutrition education periods and activities at school						
c. Nutrition education is reflected in school food environment by regulating access to unhealthy food						
d. Ensures regular training of school staff on diet/nutrition related issues						
e. Encourages staff to be role models for healthy eating						
f. Nutrition education extends beyond the school environment to parents and community						
g. Requires nutrition qualifications for school food /nutrition service staff (if applicable)						

C. Meal Environment at School

15. What type of meal programme exists in your school?
    a. There is no meal programme at school ☐
    b. Mid-day meal programme ☐
    c. School level meal programme (privately funded) ☐
    d. Others (Please specify) ________________________

16. The meal programme in your school covers which of the following classes? (Mark all that apply)
    a. Class I -V ☐
    b. Class VI-VIII ☐
    c. Class IX-X ☐
    d. Class XI-XII ☐
    e. There is no meal programme at school ☐

17. Does the meal programme at school follow any nutrient value recommendations?
    a. Yes ☐
    Please specify which recommendation/guidelines____________________
    b. No ☐
    c. There is no meal programme at school ☐

18. Which of the following are applicable to the food environment at your school?

	**Yes**				**No**	**Not applicable**
	**Primary**	**Middle**	**Higher secondary**	**Senior secondary**		
a. There are nutritional guidelines for foods available at canteen/ school meal						
b. There is monitoring of hygiene in school meal/canteen						
c. Low-fat foods and/or low-fat methods are used for preparing foods in school meal/canteen						
d. The sale of "whole foods": whole grains, unprocessed foods is promoted in school meal/canteen						
e. There is regular monitoring and revision of the school meal/ canteen menu						

	Yes				No	Not applicable
	Primary	Middle	Higher secondary	Senior secondary		
f. There is no sale of fried food in school canteen						
g. There is no sale of carbonated/sweetened beverages in school canteen						
h. There is no sale of salty foods e.g. wafers, chips etc. in school canteen						
i. There are pricing guidelines for canteens to encourage sale of healthy food choices at reduced cost						
**Lunch box/food from home**						
a. There is regulation of foods/drinks brought from home						
b. Parents are provided with written guidelines about sending healthy food in lunch boxes						
c. There is regular monitoring of lunch boxes at school						
d. There are fruits breaks at school						
**Outside food stores/vendors**						
a. Students are allowed access to food outlets around the school campus during school hours including recess						
b. There are regulations regarding the sale of food outside the school by vendors						
**Others**						
a. There are regulations regarding food menus associated with special events (e.g. sports days, annual day celebrations, birthday celebrations etc.)						
b. Unhealthy foods are used to reward students' good behavior or achievements and/or withheld as a punishment						
c. The school ensures there is adequate time for students to eat during recess						

19. Who operates the canteen service in school?
   a. There is no school canteen ☐
   b. School operates the canteen ☐
   c. Contracted to a commercial operator ☐
   d. Others, please specify ______________________________ ☐

20. Do your school level policies/rules/regulations cover the following aspects of Physical education/physical activity?

	**Yes**				**No**	**Don't Know**
	**Primary**	**Middle**	**Higher Secondary**	**Senior Secondary**		
a. Addresses physical education curriculum						
b. PE marks/grades have weightage in final examination						
c. Addresses qualifications for physical education instructors						
d. Regular Mass PT						
e. Inclusion of Yoga						
f. Yoga teacher in school						

21. Does the Physical activity environment at your school have the following aspects?

	**Yes**				**No**	**Not Applicable**
	**Primary**	**Middle**	**Higher Secondary**	**Senior Secondary**		
a. Ensures regular physical activity provision						
b. Effectively utilizes PE period by ensuring every child is physically active and engaged						
c. Promotes physical activity through intra and inter school competitions						
d. Availability of adequate and well maintained play grounds						

	Yes				No	Not Applicable
	Primary	Middle	Higher Secondary	Senior Secondary		
e. Addresses safe facilities and adequate equipment and for physical activity (eg. courts, pitches, sports goods)						
f. Addresses amount of time devoted to moderate to vigorous activity (eg. brisk walking, dancing, running etc.)						
g. Provision of budget allocation for sports equipment maintenance						
h. Addresses safe and active travel to school to promote physical activity						
i. Includes physical activity opportunities for school staff						
j. Addresses community use of school facilities for physical activity outside of the school day						

22. In the last 12 months have any sporting, social or cultural events in your school been sponsored by soft-drink, fast food or confectionary companies?
    a. Yes ☐
    b. No ☐

D. Tobacco/Alcohol

23. Does your school have any of the following policies/norms/provisions?

	Yes				No	Not Applicable
	Primary	Middle	Higher Secondary	Senior Secondary		
a. School curriculum includes information about harmful effects of tobacco						
b. School curriculum includes information about harmful effects of alcohol						

	Yes				No	Not Applicable
	Primary	Middle	Higher Secondary	Senior Secondary		
c. 23 b1 If yes, please specify the classes _______________						
d. School has provision to sensitize parents/visitors/non-teaching staff about harmful effects of tobacco use (e.g. PTMs, lectures/discussion forums, posters, resource material etc.)						
e. School has provision to sensitize parents/visitors/non-teaching staff about harmful effects of alcohol use (e.g. PTMs, lectures/discussion forums, posters, resource material etc.)						
f. School prohibits sale of tobacco inside the school campus						
g. School prohibits sale of tobacco within 100 yards of school campus						
h. School has policies/rules to prohibit smoking/tobacco use within the school campus						
i. School has policies/rules prohibit sale of alcohol around the school campus						

E. Health assessment at school

24. Does your school have a full-time, registered school nurse responsible for health services?
   a. Yes ☐
   b. No ☐
   c. Not applicable

25. Does your school provide regular health assessment at school?

   a. No ☐
   b. Yes, health assessment is done annually
      If yes, please specify the classes ___________________________________
   c. Yes, health assessment is done every 6 months ☐
      If yes, please specify the classes ___________________________________

25a. If yes, health assessment includes which of the following (mark all that apply)

a. Anthropometry (height, weight)
b. Blood group
c. Vaccination status
d. Vision
e. Oral health (teeth, mouth etc.)
f. Personal Hygiene (eyes, ear, nose, nails, skin etc.)

F. Engaging community / Communication

26. Does your school ensure distribution of information to parents about healthy food and eating?

a. Yes ☐
b. No ☐
c. Don't Know ☐

27. Are parents oriented to school health policies?

d. Yes ☐
e. No ☐
f. Don't Know ☐

28. Does your school communicate its health and safety policies in the following ways? (Mark all that apply)

a. School circular
b. Signs (e.g., tobacco-free, health promotion messages) ☐
c. Bulletin boards ☐
d. School website ☐
e. School Almanac/ Student handbook ☐
f. Staff member orientation/meetings/ employee contracts ☐
g. Student orientation at the beginning of session ☐
h. Parent handbook, newsletters ☐
i. Policies included in contracts with outside vendors/canteen ☐
j. Announcements at school events ☐
k. Parent- teachers meetings ☐

Observation Checklist

School Environment Assessment

Name of school ____

Assessment done by _____________________________ Date: _______________ Time: _____

A. Access to school

1. Number of school entrances __________
2. Number of school entrances accessible to students__________
3. For each entrance please record if there is a speed limit board/signage mentioning the speed limit (in km/ hour) on the adjacent road
   a. Yes ☐
   b. No ☐

3 a. If yes, speed limit

   i. Around main entrance ______km/hour

   ii. Around other entrance/s ______ km/hour

4. Observe and record if there are pedestrians/ walking area near the school campus
   a. Yes ☐
   b. No ☐

   If yes,____

4a. Are they accessible for convenient walking?

   i. Yes ☐

   ii. No ☐

5. Observe and record, if the following are visible from any of the school entrances.

	Yes	No
a. Place where parents can stop and drop off their children	☐	☐
b. Bus stop	☐	☐

   If yes, ____

5b 1. Distance from main entrance ______________

c. Cycle lanes:

   i. Separated from the road ☐ ☐

   ii. On the road ☐ ☐

d. A marked pedestrian crossing (e.g. zebra/ light controlled crossing) to assist access to the school ☐ ☐

e. Traffic calming measures/signages (1 if ticked; missing otherwise)

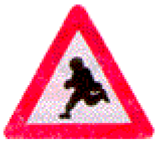
School ahead ☐

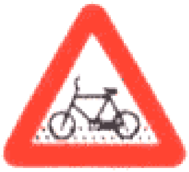
(Cycle crossing) ☐

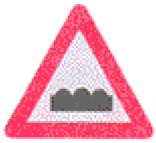
(Speed breaker) ☐

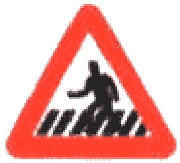
(Pedestrian crossing) ☐

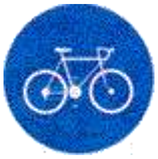
(Compulsory cycle track) ☐

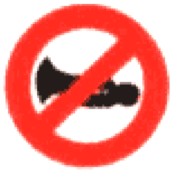
(Horn prohibited) ☐

6. Observe and record the number of designated cycle stands in the school ______________

B. The School Grounds

7. Observe and record if the school has a playground/s

   a. Yes ☐

   b. No ☐

If yes,

7a. Please specify the number of school playground/s __________

7b. Approximate measurement of each playground _______________

8. Are the school play grounds on a split site?
   a. Yes ☐
   b. No ☐

   If yes,

   8a. How do students access these playgrounds? ________________________

   8b. Are they freely accessible to students?
      i. Yes ☐
      ii. No ☐

9. Please indicate whether the following are present in the **school play area/playground** and rate their quality as good/adequate/poor :

	Number	Quality<br>Good	 Adequate	 Poor
a. Playground equipment (e.g. swings, slide etc.)		☐	☐	☐
b. Pitches (e.g. football, cricket etc.)		☐	☐	☐
c. Hard surface courts (eg. basketball court, badminton court etc.)		☐	☐	☐
c. Athletics track (grass or hard-surface)		☐	☐	☐
d. Benches		☐	☐	☐
e. Water cooler		☐	☐	☐
f. A quadrangle		☐	☐	☐
g. Others, please specify:				

10. Observe and record if any litter/animal litter remains is visible in the designated play area/play grounds?
   a. Yes ☐
   b. No ☐

11. Does the school ground have trees?
   a. Yes ☐

b. No ☐

C. Usage of School Grounds

12. Observe and record whether the school ground/s are suitable for the following

	**Very suitable**	**Somewhat suitable**	**Not suitable**
a. Sport (organized)			
b. Informal games (eg. judo, kabaddi, kho-kho, tug of war)			
c. Other outdoor games			

13. Observe the school neighborhood and record the following? (Mark all that apply)

a. Area around the school is predominantly residential ☐

b. Area around the school predominantly has open fields/parks ☐

c. Area around the school predominantly has market area/shops ☐

d. Any other ☐

13d_1. Please specify ________________________

D. Physical Education/ Physical activity

14. Number of physical education/physical activity periods / week as mentioned in time table

a. Primary (Class 1-5) _____periods/week

b. Middle (Class 6-8) _____ periods/week

c. Higher Secondary (Class 9-10) _____ periods/week

d. Senior secondary (Class 11-12) _____ periods/week

15. Observe the notice boards and walls in the school and record if there are any physical activity promoting message/posters (please take pictures with school consent only)

Number of locations with physical activity promoting messages:

a. Primary (Class 1-5) _______; located at _______________

b. Middle (Class 6-8) _______; located at _______________

c. Higher Secondary (Class 9-10) ______; located at _____________

d. Senior secondary (Class 11-12) ______ ; located at ______

16. Observe the availability of following equipment in the school:

Appropriate equipment for	Primary (Class 1-5)			Middle (Class 6-8)			Higher Secondary (Class 9-10)			Senior secondary (Class 11-12)		
	Availability Yes	No	Timings	Availability Yes	No	Timings	Availability Yes	No	Timings	Availability Yes	No	Timings
a. Swimming	☐	☐		☐	☐		☐	☐		☐	☐	
b. Table tennis	☐	☐		☐	☐		☐	☐		☐	☐	
c. Tennis court	☐	☐		☐	☐		☐	☐		☐	☐	
d. Basket ball	☐	☐		☐	☐		☐	☐		☐	☐	
e. Running track	☐	☐		☐	☐		☐	☐		☐	☐	
f. Gymnasium	☐	☐		☐	☐		☐	☐		☐	☐	
g. Field games (football, cricket etc.)	☐	☐		☐	☐		☐	☐		☐	☐	
h. Yoga	☐	☐		☐	☐		☐	☐		☐	☐	
i. Others, 16i_1. Please specify	☐	☐		☐	☐		☐	☐		☐	☐	

17. Observe the school playground and record which of the following sports are being played in the school on the day of the visit? **(Mark all that apply)**

a.None	☐	b.Tennis	☐
c.Cricket	☐	d.Cycling	☐
e.Hockey	☐	f.Kho-kho	☐
g.Football	☐	h.Kabaddi	☐
i.Gymnastics	☐	j.Swimming	☐
k.Others<br>17k_1.(Please specify)	☐		

18. Observe and record the following:

During recess

a. Teachers available in class to monitor lunchboxes ☐
b. Teachers available in and around play grounds ☐
c. Whether most children are engaged in structured or unstructured play/sport ☐
d. Whether most children are engaged in sedentary activity (eg. sit in class, chat) ☐

During PE period

e. Teachers available in class and around play grounds ☐
f. Whether most children participate in structured or unstructured play/sport ☐
g. Whether most children are engaged in sedentary activity (eg. sit in class, chat) ☐

E. Nutrition and Diet

19. There is a canteen in school Yes ☐ No ☐

19_1. Which of the following foods and drinks are available in the school canteens/cafeterias? (Mark all that apply)

**Foods**	**Drinks**
Chocolates, sweets, candies ☐	Tea/coffee ☐
Pizza ☐	Packed juices (sweetened) ☐
Burger ☐	Packed juices (no added sugar) ☐
Samosa ☐	Carbonated drinks (sweetened eg. soft drinks) ☐
Puff/Pattie ☐	Carbonated drinks (unsweetened eg. soda, diet) ☐
Chips, wafers etc. ☐	Fruit beer ☐
Fried chaat ☐	Water ☐
French fries ☐	Milk/flavored milk ☐
Noodles ☐	Lassi/flavored lassi ☐
Salads ☐	Fresh fruit juice (no added sugar) ☐
Rajma rice ☐	Others (Please specify) __________

Kadhi rice ☐	
Daal rice ☐	
Whole fruit ☐	
Others(Please specify) __________	

Any other observation in school canteen:

____

F. Vendors

20. Observe and record if there are food vendors/ hawkers selling junk food and/or carbonated drinks within 500 yards of schools campus
    a. Yes ☐
    b. No ☐

    20a. If yes, please add details____
21. During the school hours, observe and record if you see any students getting food from food outlets or venders during school hours?
    a. Yes ☐
    b. No ☐

21a. If yes, please add details ____

G. Tobacco and Alcohol

22. Observe and record if you see any tobacco vendors within a radius of 100 yards from the school campus
    a. Yes ☐
    b. No ☐
23. Observe and record if you see any alcohol outlet in the immediate vicinity of the school
    a. Yes ☐
    b. No ☐

24. Observe and record if you see anyone smoking in the school campus?

a. Yes ☐

b. No ☐

25. Observe and record if you see any tobacco litters/tobacco related products (cigarettes/bidi buds, smokeless tobacco wrappers, lighters, ashtrays etc.) in the school

a. Yes ☐

b. No ☐

H. Signage

26. Observe and record if you see any of the following signage in /outside the school campus (include all locations such as canteens, classrooms, reception area, sports room, school clinics, corridors, library, bulletin boards, office, school ground, school fences or any other)

<table>
<tr><th>Signage</th><th>Yes</th><th>No</th><th>Location</th><th>Visibility (High, medium, low)</th></tr>
<tr><td>a. Food sponsoring company</td><td>☐</td><td>☐</td><td></td><td></td></tr>
<tr><td>b. Beverage sponsoring company</td><td>☐</td><td>☐</td><td></td><td></td></tr>
<tr><td>c. School nutrition policy statement</td><td>☐</td><td>☐</td><td></td><td></td></tr>
<tr><td>d. School Physical activity policy statement</td><td>☐</td><td>☐</td><td></td><td></td></tr>
<tr><td>e. School health policy statement</td><td>☐</td><td>☐</td><td></td><td></td></tr>
<tr><td>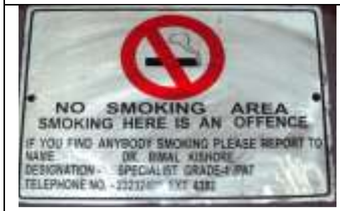
</td><td>☐</td><td>☐</td><td></td><td></td></tr>
<tr><td>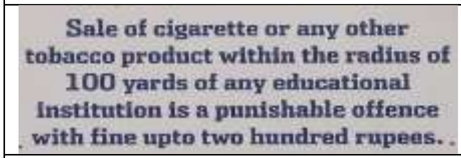
</td><td>☐</td><td>☐</td><td></td><td></td></tr>
<tr><td colspan="5">f. Others, ☐ ☐
26f_1. Please specify</td></tr>
</table>

27. Any other Observation:

____

PUBLIC HEALTH FOUNDATION OF INDIA

PARENT SURVEY - Class II

10602145

Dear Parent / Guardian,

You are invited to participate in a study entitled "*Assessing the role of school policies in emergence of non-communicable disease risk factors among school-aged children in Delhi*". This study is being conducted at your child's school by the Public Health Foundation of India (PHFI) and its partner-Health Related Information Dissemination Amongst Youth (HRIDAY) in collaboration with All India Institute of Medical Sciences (AIIMS) and the London School of Hygiene and Tropical Medicine (LSHTM). The authorities at your child's school have already agreed to participate in the study. The goal of this initiative is to come up with recommendations for school policies to make them heart healthy.

As a part of the study, we are writing to seek your permission to participate in filling of an anonymous and confidential survey. The parent survey aims to assess the dietary, physical activity, tobacco and alcohol use behaviors and their correlates as well as the family norms and practices related to these behaviours.This survey should take about 20-25 minutes to complete.Your participation in this survey is completely voluntary and you may refuse to take part, or choose to stop taking part, at any time. A decision not to take part or to stop being a part of this project will not affect your or your child's current or future relations with your child's school, PHFI, HRIDAY or any other concerned organization. You will be expected to complete the survey and return it to school along with your child within a period of two days.

All responses to the survey will be kept strictly confidential. No one-not even school staff will be able to see this information. An individual parent's response will only be seen by study staff and will not be shared with any unauthorized people. A special number will be used to identify your child and your child's school in the study and will only be seen by the project staff and will not be shared with any unauthorized people.

If you have any questions or concerns about the study/survey, please feel free to call *Dr. Monika Arora* at 011-49566000. Any of us will gladly assist you.

If you have decided to participate in this survey, please complete and sign the form below. If you do not want to participate in this survey, do NOT sign the form. Your and your child's participation in this study is very important to us so we hope you will consider taking part in it.

Thank you

Sincerely,

Monika Arora

Dr. Monika Arora

Principal Investigator & Director: Health Promotion Division

Public Health Foundation of India, New Delhi

NAME OF PARENT/GUARDIAN : ....

DETAILS OF THE CHILD

NAME OF THE CHILD : ....

NAME OF THE SCHOOL : ....

TYPE OF SCHOOL : Government ☐ Private ☐

CLASS AND SECTION : ....

GENDER : Boy ☐ Girl ☐

STATE : Delhi ☐ Haryana ☐

DATE OF BIRTH : ☐☐ (Day) ☐☐ (Month) ☐☐☐☐ (Year)

AGE (COMPLETED YEARS) : ....

TODAY'S DATE : ☐☐ ☐☐ 2 0 ☐☐

YOUR ADDRESS : ....

....

SCJ - 1997

SIGNATURE OF THE PARENT / GUARDIAN : ....

10602145

PARENT SURVEY - Class II

Instructions :

- This questionnaire you are about to complete is important. The information you share with us will be used to develop health and nutrition programmes for children and youth.
- Please read each question and its options carefully before answering.
- Choose the most appropriate answer according to you.
- For every question or its sub-parts, choose only one answer (unless specified) by filling the bubble (●).
- Please only use the pencil provided to you to fill the bubbles.
- If you do not understand a question, please ask for a help.
- Do not spend too much time on any one question. The questionnaire should take you around 20-25 minutes to complete.
- There is no right or wrong answer. Please be as honest as you can with all of your responses.

A. BACKGROUND INFORMATION

1. Highest education attained by child's mother

- a. Professional or higher degree (MPhil., PhD) ○
- b. Graduate or post graduate (B.A/B.Sc./B.Ed./MBBS/M.A/M.Ed. etc.) ○
- c. Intermediate (Class 12th) or post high school diploma ○
- d. High school certificate (Class 10th) ○
- e. Middle school certificate (Class 8th) ○
- f. Primary school certificate (Class 5th) ○
- g. Illiterate (No formal education) ○

2. Highest education attained by child's father / guardian:

- a. Professional or higher degree (MPhil., PhD) ○
- b. Graduate or post graduate (B.A/B.Sc./B.Ed./MBBS/M.A/M.Ed. etc.) ○
- c. Intermediate (Class 12th) or post high school diploma ○
- d. High school certificate (Class 10th) ○
- e. Middle school certificate (Class 8th) ○
- f. Primary school certificate (Class 5th) ○
- g. Illiterate (No formal education) ○

3. Occupation of child's mother

- a. Professional (e.g. doctors, nurses, lawyers, engineers, teachers etc.) ○
- b. Semi-Professional (technicians, assistants etc.) ○
- c. Clerical, Shop-owner, farmer ○
- d. Skilled worker (with formal training or certificate) ○
- e. Semi-skilled worker (without any formal training or certificate) ○
- f. Unskilled worker (labourer) ○
- g. Unemployed / homemaker ○

4. Occupation of child's father / guardian

- a. Professional (e.g. doctors, nurses, lawyers, engineers, teachers etc.) ○
- b. Semi-Professional (technicians, assistants etc.) ○
- c. Clerical, Shop-owner, farmer ○
- d. Skilled worker (with formal training or certificate) ○
- e. Semi-skilled worker (without any formal training or certificate) ○
- f. Unskilled worker (labourer) ○
- g. Unemployed ○

5. What is your monthly family income from all sources?

- a. Rs. 1520 or less ○
- b. Rs. 1521-4555 ○
- c. Rs. 4556-7593 ○
- d. Rs. 7594-11,361 ○
- e. Rs. 11,362-15,187 ○
- f. Rs. 15,188-30,374 ○
- g. Rs. 30,375 or more ○

B. KNOWLEDGE

Diet / Nutrition

6. Energy balance is achieved when over time

- a. Energy In (food intake) is greater than Energy Out (physical activity) ○
- b. Energy In (food intake) is less than Energy Out (physical activity) ○
- c. Energy In (food intake) is equal to Energy Out (physical activity) ○
- d. All of the above ○
- e. None of the above ○
- f. Don't know ○

7. Which of the following are important food groups for a balanced diet? (Mark all that apply)

- a. Cereals (e.g. Brown Bread, Porridge etc.) ○
- b. Pulses / Meat / eggs ○
- c. Fruits and vegetables ○
- d. Milk and milk products ○
- e. Sugar and oil ○
- f. Don't know ○

8. Based on your knowledge answer the following:

	Yes	No	Don't know
a. Is skipping meals a good way to lose weight?	○	○	○
b. Can skipping breakfast lead to overweight / obesity?	○	○	○
c. Can excessive intake of salty snacks (e.g. wafers, chips, namkeen etc.) lead to high blood pressure?	○	○	○
d. Is consumption of fruit juices better than whole fruits?	○	○	○
e. Can watching TV while eating lead to overweight/obesity?	○	○	○

Physical Activity

9. Do you agree with the following:

	Yes	No	Don't know
a. It is important to include physical activity in daily routine to maintain healthy weight	○	○	○
b. Taking part in physical activities can help in getting better grades/marks at school	○	○	○
c. The only way to include physical activity in the daily routine is to play sports	○	○	○
d. Being physically inactive (sedentary) can increase the risk of heart diseases	○	○	○

10. As a part of a healthy lifestyle, how many hours a day should your child spend watching TV, DVD, playing video games, mobile games or computer games?

a. Less than 2 hours per day ○
b. 2-3 hours per day ○
c. 3-4 hours per day ○
d. 4-5 hours per day ○
e. More than 5 hours per day ○
f. Don't know ○

11. The minimum amount of time recommended for moderate to vigorous physical activity daily (running, cycling, brisk walking, jogging, play sports etc.) for healthy living among children and youth is:

a. 15 minutes per day ○
b. 30 minutes per day ○
c. 45 minutes per day ○
d. 60 minutes per day ○
e. Don't know ○

12. Lack of regular physical activity may lead to the following: (Mark all that apply)

a. Overweight / Obesity ○
b. Diabetes ○
c. Osteoporosis (porous bones with increased risk of fracture) ○
d. None of the above ○
e. Don't know ○

Tobacco

[Tobacco can be consumed in many forms. It can be smoked, such as cigarette smoking or bidi smoking or hookah. It can also be chewed, such as gutkha and khaini]

13. Based on what you know, can tobacco use cause the following diseases? (Mark all that apply)

a. Heart Attack ○
b. Lung Cancer ○
c. Tuberculosis ○
d. Oral Cancer ○
e. None of the above ○
f. Don't know ○

14. Based on what you know, can smoking around non-smokers increase risk of illness among non-smokers?

a. Yes ○
b. No ○
c. Don't know ○

15. Based on what you know, does your state have law which prohibits the following (Mark all that apply):

a. Sale of tobacco products to minors (under 18 years of age) ○
b. Sale of tobacco products within 100 yards of your child's school ○
c. Advertising tobacco products within and around your child's school campus ○
d. Smoking within the premises of your child's school ○
e. None of the above ○
f. Don't know ○

Alcohol

16. Based on what you know, can excessive alcohol consumption lead to any of the following diseases? (Mark all that apply)

a. Heart Attack ○
b. Hypertension (high blood pressure) ○
c. Liver Disease ○
d. Anxiety / depression ○
e. None of the above ○
f. Don't know ○

17. Based on what you know, can alcohol consumption lead to any of the following behaviours? (Mark all that apply)

a. Violence ○
b. Injury/ accidents ○
c. Social issues like harassment, eve teasing etc. ○
d. None of the above ○
e. Don't know ○

18. The minimum age at which one can consume alcohol in your state (minimum legal drinking age) is:

a. 18 years ○
b. 21 years ○
c. 25 years ○
d. None of the above ○
e. Don't know ○

19. Is alcohol consumption or sale allowed in the area around your child's school?

a. Yes ○
b. No ○
c. Don't know ○

10602145

C. BEHAVIOUR OF YOUR CHILD

Diet / Nutrition

20. Is your child :

a. Vegetarian ◯
b. Non vegetarian ◯
c. Ovo-vegetarian (vegetarian with only egg) ◯

21. On most of the days, how many times does your child eat/drink the following foods?

	Never	Ocassionally (less than once a day e.g. once a week or once every few weeks)	Once a day	2 times/day	3 or more times/day
a. Whole fruits (do not count fruit juices)	◯	◯	◯	◯	◯
b. Vegetables (do not count salad and potatoes)	◯	◯	◯	◯	◯
c. Salad (e.g. lettuce, tomato, cucumber, etc.)	◯	◯	◯	◯	◯
d. Whole grains (e.g. chapatti, brown bread, brown rice etc.)	◯	◯	◯	◯	◯
e. Pulses/lentils (e.g. rajma, chole, chana, moong dal etc.)	◯	◯	◯	◯	◯
f. Milk, curd, buttermilk, yoghurt	◯	◯	◯	◯	◯

22. In a week, how often does your child eat the following foods?

	Never	1-2 times / week	3-4 times / week	5-6 times / week	7 times / week	More than 7 times/week
a. Deep fried foods (e.g. samosa, pakoda, bread pakora, kachori, vada, puri etc.)	◯	◯	◯	◯	◯	◯
b. Fast/Junk food (e.g. Burger, Pizza, noodles etc.)	◯	◯	◯	◯	◯	◯
c. Fruit juices (e.g. orange, apple etc.)	◯	◯	◯	◯	◯	◯
d. Soft drinks / aerated beverages	◯	◯	◯	◯	◯	◯
e. Squashes	◯	◯	◯	◯	◯	◯
f. Chips, wafers, namkeen	◯	◯	◯	◯	◯	◯
g. Chocolates, candies, toffees	◯	◯	◯	◯	◯	◯
h. Dessert (eg. kheer, halwa, jalebi, kulfi, Ice-cream etc.)	◯	◯	◯	◯	◯	◯
i. Doughnuts, pastry, cake, muffin etc.	◯	◯	◯	◯	◯	◯

23. In a week, on how many days does your child eat breakfast?

a. Never ◯
b. 1-2 days ◯
c. 3-4 days ◯
d. 5-6 days ◯
e. Every day ◯

24. On school days, where does your child usually get food for his/her lunch break?

a. My child does not eat anything at school ◯
b. School canteen ◯
c. Hawkers / rehri wala ◯
d. Tiffin box from home ◯
e. Mid-Day Meal from school ◯
f. Others, Please specify________________ ◯

25. How often do you read the nutritional label of packed food items while purchasing them for your child?

a. Most of the times ◯
b. Sometimes ◯
c. Rarely ◯
d. Never ◯

26. How often does your child eat food / snack while watching TV?

a. Most of the times ◯
b. Sometimes ◯
c. Rarely ◯
d. Never ◯

27. Does your child eat more than usual when he/she is:

	Surely Yes	May be Yes	May be No	Surely No
a. Out with friend/s	◯	◯	◯	◯
b. Out with family	◯	◯	◯	◯
c. Studying for exams	◯	◯	◯	◯
d. Physically active	◯	◯	◯	◯
e. Bored	◯	◯	◯	◯
f. Angry	◯	◯	◯	◯
g. Sad	◯	◯	◯	◯
h. Stressed out (other than studies)	◯	◯	◯	◯
i. While watching movie/Television	◯	◯	◯	◯

Physical Activity

28. On most of the days, how does your child travel from home to school and school to home? (Select all that apply)

	Yes	No	If Yes, Please specify duration (in minutes)
a. Walking	◯	◯	
b. Cycling	◯	◯	
c. Public transport (Bus, metro, auto etc.)	◯	◯	
d. Private vehicle (school bus, car, two wheeler etc.)	◯	◯	

29. What kind of activities does your child perform during his/her physical education / PT period in school?(Mark all that apply)

- a. Outdoor sports which involves running (e.g. football, basketball) ○
- b. Indoor games ○
- c. PT / drill ○
- d. Yoga ○
- e. He / she does not participate ○
- f. Others, please specify ____________ ○
- g. Don't know ○

30. On school days, what does your child do most of the time during his or her lunch break (apart from eating)?

- a. Mostly sits down, chats with friends ○
- b. Mostly played inside the class ○
- c. Mostly played active games outside the class ○
- d. Complets homework ○
- e. Other, please specify ____________ ○
- f. Don't know ○

31. Do all students have access to sports equipment available in your child's school?

- a. Yes, free access to all students during Physical education / physical activity period ○
- b. Yes, free access to all students during recess ○
- c. No, limited access (to students participating in school's sports team only) ○
- d. No, limited access (due to shortage of equipment) ○
- e. There are no sports equipment in the school ○
- f. Don't know ○

32. In a week, on how many days is your child involved in extra - curricular activities at school which require being physically active (e.g. dancing, playing games etc.)?

- a. Never ○
- b. 1-2 days ○
- c. 3-4 days ○
- d. Every day ○
- e. Don't know ○

33. On most of the days, how many hours does your child do the following in his/her leisure time?

	Never	Less than 1 hour/day	1-2 hours/day	2-3 hours/day	3-4 hours/day	More than 4 hours/day
a. Watch TV on a school day (Mon – Fri)	○	○	○	○	○	○
b. Watch TV on weekend (Sat – Sun)	○	○	○	○	○	○
c. Play video games on a school day (Mon – Fri)	○	○	○	○	○	○
d. Play video games on a weekend day (Sat – Sun)	○	○	○	○	○	○
e. Use computers (other than for doing homework) on a school day (Mon–Fri)	○	○	○	○	○	○
f. Use computers (other than for doing homework) on a weekend day (Sat–Sun)	○	○	○	○	○	○
g. Studying/tuition on a school day (Mon-Fri)	○	○	○	○	○	○
h. Studying / tuition on a weekend day (Sat – Sun)	○	○	○	○	○	○

34. On most of the days, how many hours does your child spend doing the following activities:

	Never	Upto 30 minutes/days	1 hour/day	2 hours/day	3 hours/day	4 or more than 4 hours/day
a. Vigorous physical activity (e.g: running, fast cycling, fast swimming or moving heavy loads)	○	○	○	○	○	○
b. Moderate physical activity (not exhausting e.g. brisk walking, dancing etc)	○	○	○	○	○	○
c. Mild physical activities (little effort e.g. walking slowly (to school, friend's house, yoga etc.)	○	○	○	○	○	○

D. INTENTIONS

Nutrition and physical activity

35. How often your child is likely to do the following?

	Never	Sometimes	Often	Very Often
a. Eat healthy food	○	○	○	○
b. Refuse /avoid junk food	○	○	○	○
c. Stay fit and exercise	○	○	○	○

E. PERCEPTION

Nutrition

36. How important are the following for you?

	Very Important	Important	Not Important	Not at all Important
a. Your child eats healthy food	○	○	○	○
b. Your child exercises and stays fit	○	○	○	○
c. Your child maintains healthy weight	○	○	○	○
d. Your child performs well in school	○	○	○	○
e. Your child participates and performs well in sports	○	○	○	○
f. Your child eats at least one meal a day with family	○	○	○	○

37. How satisfied are you with your child's:

	Very Satisfied	Satisfied	Not Satisfied	Not at all Satisfied
a. Height	○	○	○	○
b. Weight	○	○	○	○

10602145

38. Does any food or beverage advertisement seem attractive to your child?

a. Yes ○

b. No ○

38. (a). If yes, please specify the brand of advertisement which influences your child's demand/purchasing behaviour

a. Food ______________

b. Beverage ______________

Physical Activity

39. Do you think that increasing facilities in your child's school (sports equipment / improving school grounds/improved supervision etc.) will help your child remain physically active?

a. Surely Yes ○

b. Maybe Yes ○

c. Maybe No ○

d. Surely No ○

40. Do you think that increasing Physical education/physical activity period will help your child remain physically active?

a. Surely Yes ○

b. Maybe Yes ○

c. Maybe No ○

d. Surely No ○

41. Do you think giving marks/grades for Physical education/physical activity subjects may improve physical activity levels among students of your child's age?

a. Surely Yes ○

b. Maybe Yes ○

c. Maybe No ○

d. Surely No ○

F. FAMILY NORMS, PRACTICE & SUPPORT

42. How often are you likely to do the following:

	Once in a while	*Most of the times*	*All the time*	*Never*
a. Limit your child's intake of sweets and sugary foods	○	○	○	○
b. Limit your child's intake of drinking soft drinks	○	○	○	○
c. Encourage your child to eat healthy foods such as fruits, vegetables, whole grains	○	○	○	○
d. Eat meals with your child	○	○	○	○
e. Encourage your child to be physically active	○	○	○	○
f. Allow your child to watch TV	○	○	○	○
g. Physically active with your child	○	○	○	○
h. Watch TV with your child	○	○	○	○
i. Smoke at home	○	○	○	○
j. Chew tobacco at home	○	○	○	○
k. Consume alcohol at home	○	○	○	○

43. Does your child have a TV or computer in his / her bedroom?

a. Yes ○

b. No ○

44. During the school days, do you limit how long your child watches TV?

a. No limits ○

b. Yes, but not very strict limits ○

c. Yes, strict limits ○

d. He / she doesn't get much time for watching TV ○

45. During the school days, do you limit how long your child uses the computer (other than for doing homework, project work etc.)?

a. No limits ○

b. Yes, but not very strict limits ○

c. Yes, strict limits ○

d. He / she doesn't get much time for using computer / net surfing ○

46. Do you or any of your family members (staying with you in your house) smoke or chew tobacco in any form (such as cigarettes, bidis, or gutkha)?

a. Yes ○

b. No ○

47. Do you or any of your family members (staying with you in your house) drink any type of alcohol?

a. Yes ○

b. No ○

G. SCHOOL ENVIRONMENT

48. Which of the following are accessible to your child during school hours at his/her school (Mark all that apply):

a. Mid-day meal ○

b. Canteen in school campus ○

c. Food vendors/shops around school campus ○

d. Playground ○

e. Sport or exercise equipment ○

f. Other Sports facilities (Swimming pool, running track in school campus) ○

g. Yoga classes ○

49. In a week, how many Physical Education/Physical Activity periods does your child have?

a. None ○

b. 1 per week ○

c. 2-4 per week ○

d. Everyday ○

50. Are students given marks/ grades for physical education subjects (including practical) in your child's school?

a. Yes ○

b. No ○

51. Is there a canteen in your child's school

a. Yes ○
b. No ○

52. Do you know what items are sold in your child's school canteen

a. There is no canteen in my child's school ○
b. Yes ○
C. No ○

52. (a) If yes, which of the following foods and drinks are available in your child's school canteens/cafeterias? (Mark all that apply)

Foods	Drinks
a. Chocolates, sweets, candies ○	a. Tea/coffee ○
b. Pizza ○	b. Packed juices (sweetened) ○
c. Burger ○	c. Packed juices (no added sugar) ○
d. Samosa ○	d. Carbonated drinks (sweetened eg. soft drinks) ○
e. Puff/Pattie ○	e. Carbonated drinks (unsweetened eg. soda, diet) ○
f. Chips, wafers etc. ○	f. Fruit beer ○
g. Fried chaat ○	g. Water ○
h. French fries ○	h. Milk / flavored mil[illegible] ○
i. Noodles ○	i. Lassi / flavored [illegible]ssi ○
j. Salads ○	j. Fresh fruit juice (no added sugar) ○
k. Rajma rice ○	k. Others (Please specify) ○ ________
l. Kadhi rice ○	
m. Daal rice ○	
n. Whole fruit ○	
o. Others (Please specify) ○ ________	

53. Do you think the foods served in your child's school canteen are?

a. There is no canteen in my child's school ○
b. Healthy ○
c. Unhealthy ○
d. Both healthy and unhealthy ○
e. Don't know ○

54. Does your child's school canteen have restriction on the sale of any particular food items/drinks?

a. There is no canteen in my child's school ○
b. Yes ○
c. No ○
d. Don't know ○
e. If yes, please specify ________ ○

55. Does your child's teacher/assigned person/class monitor check the food (lunchboxes) brought from home?

a. Yes ○
b. No ○
c. Don't know ○

55. (a) If yes, do they give / write instructions for you (as parent) to provide healthy food to your child ?

a. Yes ○
b. No ○

56. Does your child's school have any of the following display boards/ signage at the school entrance or in the school campus?

I. "Tobacco free school" board at a prominent place of the school entrance

a. Yes ○
b. No ○
c. Don't know ○

II. "No smoking area – smoking here is an offence" inside the school

a. Yes ○
b. No ○
c. Don't know ○

57. Have you ever observed sale of tobacco within 100 yards of your child's school campus?

a. Yes ○
b. No ○

58. Have you ever observed sale of alcohol within 75 meters in the vicinity of your child's school campus?

a. Yes ○
b. No ○

Thank you for filling this questionnaire.
Your help with this project is greatly appreciated.

STUDENT SURVEY - Class XI

PUBLIC HEALTH FOUNDATION OF INDIA

10802110

Dear Student,

You are invited to participate in a study entitled *"Assessing the role of school policies in emergence of non-communicable disease risk factors among school-aged children in Delhi"*. This study is being conducted at your school by the Public Health Foundation of India (PHFI) and its partner-Health Related Information Dissemination Amongst Youth (HRIDAY) in collaboration with All India Institute of Medical Sciences (AIIMS) and the London School of Hygiene and Tropical Medicine (LSHTM). The authorities at your school have already agreed to participate in the study. The goal of this initiative is to come up with recommendations for school policies to make them heart healthy.

This study involves filling of an anonymous and confidential survey in class. The survey aims to assess the dietary, physical activity, tobacco and alcohol use behaviours and their correlates and practices related to these behaviours. This survey should take about 35-40 minutes to complete. A decision not to take part or to stop being a part of this project will not affect your current or future relations with your school, PHFI, HRIDAY or any other concerned organization. If you do not participate in the survey administered during class, the teacher will provide an assignment for you to work on while the other students are participating.

All responses to the survey will be kept strictly confidential. No one-not even your school staff, parents and friends will be able to see this information. An individual student's response will only be seen by study staff and will not be shared with any unauthorized people. A special number will be used to identify you and your school in the study and will only be seen by the project staff and will not be shared with any unauthorized people.

If you have any questions or concerns about the survey, please feel free to call *Dr. Monika Arora* at 011-49566000. Any of us will gladly assist you.

If you have decided to participate in this survey, please complete and sign the form below. If you do not want to participate in this survey, do NOT sign the form.Your participation in this study is very important to us so we hope you will consider taking part in it.

Thank you

Sincerely,

Monika Arora

Dr. Monika Arora
Principal Investigator & Director: Health Promotion Division
Public Health Foundation of India, New Delhi

DETAILS OF THE CHILD

NAME OF THE CHILD : ....

NAME OF THE SCHOOL : ....

TYPE OF SCHOOL : Government ☐ Private ☐

CLASS AND SECTION : ....

GENDER : Boy ☐ Girl ☐

STATE : Delhi ☐ Haryana ☐

YOUR BIRTHDATE HERE : ☐☐ (Day) ☐☐ (Month) ☐☐☐☐ (Year)

AGE (COMPLETED YEARS) : ....

TODAY'S DATE HERE : ☐☐ ☐☐ 2 0 ☐☐

YOUR ADDRESS : ....

....

SIGNATURE OF THE STUDENT : ....

SCJ - 1998

STUDENT SURVEY - Class XI

10802110

Instructions :

- This questionnaire you are about to complete is important. The information you share with us will be used to develop health and nutrition programmes for children and youth.
- Please read each question and its options carefully before answering.
- Choose the most appropriate answer according to you.
- For every question or its sub-parts, choose only one answer (unless specified) by filling the bubble (⬬).
- Please only use the pencil provided to you to fill the bubbles.
- If you do not understand a question please ask for a help.
- Do not spend too much time on any one question. The questionnaire should take you around 35-40 minutes to complete.
- There is no right or wrong answer. Please be as honest as you can with all of your responses.

A. BACKGROUND INFORMATION

1. Highest education attained by your mother:
   a. Professional or higher degree (MPhil., PhD) ◯
   b. Graduate or post graduate (B.A/B.Sc./B.Ed./MBBS/M.A/M.Ed. etc.) ◯
   c. Intermediate (Class 12th) or post high school diploma ◯
   d. High school certificate (Class 10th) ◯
   e. Middle school certificate (Class 8th) ◯
   f. Primary school certificate (Class 5th) ◯
   g. Illiterate (No formal education) ◯

2. Highest education attained by your father / guardian:
   a. Professional or higher degree (MPhil., PhD) ◯
   b. Graduate or post graduate (B.A/B.Sc./B.Ed./MBBS/M.A/M.Ed. etc.) ◯
   c. Intermediate (Class 12th) or post high school diploma ◯
   d. High school certificate (Class 10th) ◯
   e. Middle school certificate (Class 8th) ◯
   f. Primary school certificate (Class 5th) ◯
   g. Illiterate (No formal education) ◯

3. Your mother's occupation:
   a. Professional (e.g. doctors, nurses, lawyers, engineers, teachers etc.) ◯
   b. Semi-Professional (technicians, assistants etc.) ◯
   c. Clerical, Shop-owner, farmer ◯
   d. Skilled worker (with formal training or certificate) ◯
   e. Semi-skilled worker (without any formal training or certificate) ◯
   f. Unskilled worker (labourer) ◯
   g. Unemployed / homemaker ◯

4. Your father's/guardian occupation:
   a. Professional (e.g. doctors, nurses, lawyers, engineers, teachers etc.) ◯
   b. Semi-Professional (technicians, assistants etc.) ◯
   c. Clerical, Shop-owner, farmer ◯
   d. Skilled worker (with formal training or certificate) ◯
   e. Semi-skilled worker (without any formal training or certificate) ◯
   f. Unskilled worker (labourer) ◯
   g. Unemployed ◯

5. What is your monthly family income from all sources?
   a. Rs. ≤ 1520 ◯
   b. Rs. 1521-4555 ◯
   c. Rs. 4556-7593 ◯
   d. Rs. 7594-11,361 ◯
   e. Rs. 11,362-15,187 ◯
   f. Rs. 15,188-30,374 ◯
   g. Rs. ≥ 30,375 ◯

B. KNOWLEDGE

Diet / Nutrition

6. Energy balance is achieved when over time
   a. Energy In (food intake) is greater than Energy Out (physical activity) ◯
   b. Energy In (food intake) is less than Energy Out (physical activity) ◯
   c. Energy In (food intake) is equal to Energy Out (physical activity) ◯
   d. All of the above ◯
   e. None of the above ◯
   f. Don't know ◯

7. Which of the following are important food groups for a balanced diet? (Mark all that apply)
   a. Cereals (e.g. Brown Bread, Porridge etc.) ◯
   b. Pulses / Meat / eggs ◯
   c. Fruits and vegetables ◯
   d. Milk and milk products ◯
   e. Sugar and oil ◯
   f. Don't know ◯

8. Based on your knowledge answer the following:

	Yes	No	Don't know
a. Is skipping meals a good way to lose weight?	◯	◯	◯
b. Can skipping breakfast lead to overweight / obesity?	◯	◯	◯
c. Can excessive intake of salty snacks (e.g. wafers, chips, namkeen etc.) lead to high blood pressure?	◯	◯	◯
d. Is consumption of fruit juices better than whole fruits?	◯	◯	◯
e. Can watching TV while eating lead to overweight/obesity?	◯	◯	◯

Physical Activity

9. Do you agree with the following:

	Yes	No	Don't know
a. It is important to include physical activity in daily routine to maintain healthy weight	◯	◯	◯
b. Taking part in physical activities can help in getting better grades/marks at school	◯	◯	◯
c. The only way to include physical activity in the daily routine is to play sports	◯	◯	◯
d. Being physically inactive (sedentary) can increase the risk of heart diseases	◯	◯	◯

10. As a part of a healthy lifestyle, how many hours a day should you spend watching TV, DVD, playing video games, mobile games or computer games?

- a. Less than 2 hours per day ◯
- b. 2-3 hours per day ◯
- c. 3-4 hours per day ◯
- d. 4-5 hours per day ◯
- e. More than 5 hours per day ◯
- f. Don't know ◯

11. The minimum amount of time recommended for moderate to vigorous physical activity daily (running, cycling, brisk walking, jogging, play sports etc.) for healthy living among children and youth is:

- a. 15 minutes per day ◯
- b. 30 minutes per day ◯
- c. 45 minutes per day ◯
- d. 60 minutes per day ◯
- e. Don't know ◯

12. Lack of regular physical activity may lead to the following: (Mark all that apply)

- a. Overweight / Obesity ◯
- b. Diabetes ◯
- c. Osteoporosis (porous bones with increased risk of fracture) ◯
- d. None of the above ◯
- e. Don't know ◯

Tobacco

[Tobacco can be consumed in many forms. It can be smoked, such as cigarette smoking or bidi smoking or hookah. It can also be chewed, such as gutkha and khaini]

13. Based on what you know, can tobacco use cause the following diseases? (Mark all that apply)

- a. Heart Attack ◯
- b. Lung Cancer ◯
- c. Tuberculosis ◯
- d. Oral Cancer ◯
- e. None of the above ◯
- f. Don't know ◯

14. Based on what you know, can smoking around non-smokers increase risk of illness among non-smokers?

- a. Yes ◯
- b. No ◯
- c. Don't know ◯

15. Based on what you know, does your state have law which prohibits the following (Mark all that apply):

- a. Sale of tobacco products to minors (under 18 years of age) ◯
- b. Sale of tobacco products within 100 yards of your school ◯
- c. Advertising tobacco products within and around your school campus ◯
- d. Smoking within the premises of your school ◯
- e. None of the above ◯
- f. Don't know ◯

Alcohol

16. Based on what you know, can excessive alcohol consumption lead to any of the following diseases? (Mark all that apply)

- a. Heart Attack ◯
- b. Hypertension (high blood pressure) ◯
- c. Liver Disease ◯
- d. Anxiety / depression ◯
- e. None of the above ◯
- f. Don't know ◯

17. Based on what you know, can alcohol consumption lead to any of the following behaviours? (Mark all that apply)

- a. Violence ◯
- b. Injury / accidents ◯
- c. Social issues like harassment, eve teasing etc. ◯
- d. None of the above ◯
- e. Don't know ◯

18. The minimum age at which one can consume alcohol in your state (minimum legal drinking age) is:

- a. 18 years ◯
- b. 21 years ◯
- c. 25 years ◯
- d. None of the above ◯
- e. Don't know ◯

19. Is alcohol consumption or sale allowed in the area around your school?

- a. Yes ◯
- b. No ◯
- c. Don't know ◯

10802110

C. BEHAVIOUR

Diet / Nutrition

20. Are you a:

a. Vegetarian ○
b. Non vegetarian ○
c. Ovo- vegetarian (vegetarian with only egg) ○

21. On most of the days, how many times do you eat/drink following foods?

	Never	Ocassionally (less than once a day e.g. once a week or once every few weeks)	Once a day	2 times/day	3 or more times/day
a. Whole fruits (do not count fruit juices)	○	○	○	○	○
b. Vegetables (do not count salad and potatoes)	○	○	○	○	○
c. Salad (e.g. lettuce, tomato, cucumber, etc.)	○	○	○	○	○
d. Whole grains (e.g. chapatti, brown bread, brown rice etc.)	○	○	○	○	○
e. Pulses/lentils (e.g. rajma, chole, chana, moong dal etc.)	○	○	○	○	○
f. Milk, curd, buttermilk, yoghurt	○	○	○	○	○

22. In a week, how often do you eat the following foods?

	Never	1-2 times / week	3-4 times / week	5-6 times / week	7 times / week	More than 7 times/week
a. Deep fried foods (e.g. samosa, pakoda, bread pakora, kachori, vada, puri etc.)	○	○	○	○	○	○
b. Fast/Junk food (e.g. Burger, Pizza, noodles etc.)	○	○	○	○	○	○
c. Fruit juices (e.g. orange, apple etc.)	○	○	○	○	○	○
d. Soft drinks / aerated beverages	○	○	○	○	○	○
e. Squashes	○	○	○	○	○	○
f. Chips, wafers, namkeen	○	○	○	○	○	○
g. Chocolates, candies, toffees	○	○	○	○	○	○
h. Dessert (eg. kheer, halwa, jalebi, kulfi, Ice-cream etc,.)	○	○	○	○	○	○
i. Doughnuts, pastry, cake, muffin etc.	○	○	○	○	○	○

23. In a week, on how many days do you eat breakfast?

a. Never ○
b. 1-2 days ○
c. 3-4 days ○
d. 5-6 days ○
e. Every day ○

24. On school days, where do you usually get food for your lunch break?

a. I don't eat anything at school ○
b. School canteen ○
c. Hawkers / rehri wala ○
d. Tiffin box from home ○
e. Mid-Day Meal from school ○
f. Others, Please specify__________ ○

25. How often do you read the nutritional label of packed food items while purchasing/eating them?

a. Most of the times ○
b. Sometimes ○
c. Rarely ○
d. Never ○

26. How often do you eat food/snack while watching TV?

a. Most of the times ○
b. Sometimes ○
c. Rarely ○
d. Never ○

27. Do you eat more than usual when you are:

	Surely Yes	May be Yes	May be No	Surely No
a. Out with friend/s	○	○	○	○
b. Out with family	○	○	○	○
c. Studying for exams	○	○	○	○
d. Physically active	○	○	○	○
e. Bored	○	○	○	○
f. Angry	○	○	○	○
g. Sad	○	○	○	○
h. Stressed out (other than studies)	○	○	○	○
i. While watching movie/Television	○	○	○	○

28. In the past one year, have you done anything to reduce weight?

a. Yes ○
b. No ○

28. (a). If Yes, (Mark all that apply)

a. Increased duration and intensity of physical activity ○
b. Decreased consumption of fried food / sugar ○
c. Increased consumption of fruits and vegetables ○
d. Skip meals ○
e. Medicines and other remedies ○
f. Others, Please specify__________ ○

Physical Activity

29. On most of the days, how do you commute from home to school and school to home? (Select all that apply)

	Yes	No	If Yes, Pleases specify duration (in minutes)
a. Walking	○	○	
b. Cycling	○	○	
c. Public transport (Bus, metro, auto etc.)	○	○	
d. Private vehicle (school bus, car, two wheeler etc.)	○	○	

30. What kind of activities do you perform during your physical education / PT period in school?(Mark all that apply)

- a. Outdoor sports which involves running (e.g. football, basketball) ○
- b. Indoor games ○
- c. PT / drill ○
- d. Yoga ○
- e. I do not participate ○
- f. Others, please specify ________________ ○

31. Over the last 5 school days, what did you do most of the time during lunch break (apart from eating)?

- a. Mostly sat down, chats with friends ○
- b. Mostly played inside the class ○
- c. Mostly played active games outside the class ○
- d. Completed homework / Studied ○
- e. Other, please specify ________________ ○

32. Do all students have access to sports equipment available in your school ?

- a. Yes, free access to all students during Physical education / physical activity period ○
- b. Yes, free access to all students during recess ○
- c. No, limited access (to students participating in school's sports team only) ○
- d. No, limited access (due to shortage of equipment) ○
- e. There are no sports equipment in the school ○
- f. Don't know ○

33. In a week, on how many days were you involved in extra - curricular activities at school which require being physically active (e.g. dancing, playing games, gymnastics etc.)?

- a. Never ○
- b. 1-2 days ○
- c. 3-4 days ○
- d. Every day ○
- e. Don't know ○

34. On most of the days, how many hours do you do the following in your leisure time?

	Never	Less than 1 hour/day	1-2 hours/day	2-3 hours/day	3-4 hours/day	More than 4 hours/day
a. Watch TV on a school day (Mon – Fri)	○	○	○	○	○	○
b. Watch TV on weekend (Sat – Sun)	○	○	○	○	○	○
c. Play video games on a school day (Mon – Fri)	○	○	○	○	○	○
d. Play video games on a weekend day (Sat – Sun)	○	○	○	○	○	○
e. Use computers (other than for doing homework) on a school day (Mon–Fri)	○	○	○	○	○	○
f. Use computers (other than for doing homework) on a weekend day (Sat–Sun)	○	○	○	○	○	○
g. Studying / tuition on a school day (Mon-Fri)	○	○	○	○	○	○
h. Studying / tuition on a weekend day (Sat – Sun)	○	○	○	○	○	○

35. On most of the days, how many hours do you spend doing the following activities?

	Never	Upto 30 minutes/days	1 hour/day	2 hours/day	3 hours/day	4 or more than 4 hours/day
a. Vigorous physical activity (e.g: running, fast cycling, fast swimming or moving heavy loads)	○	○	○	○	○	○
b. Moderate physical activity (not exhausting e.g. brisk walking, dancing etc)	○	○	○	○	○	○
c. Mild physical activities (little effort e.g. walking slowly (to school, friend's house, yoga etc.)	○	○	○	○	○	○

Tobacco Use Behaviour

36. Have you ever chewed tobacco in any form (eg. Guthka or Khani) ?

- a. Yes ○
- b. No ○

36. (a). If yes, how old were you when you first chewed tobacco?

__________ Years

37. Have you chewed tobacco during the past 30 days?

- a. Yes ○
- b. No ○

38. Have you ever smoked tobacco in any form?

- a. Yes ○
- b. No ○

38. (a). If yes, how old were you when you first smoked tobacco?

__________ Years

39. Have you smoked tobacco during the past 30 days?

- a. Yes ○
- b. No ○

10802110

40. How many of your friends smoke or chew tobacco in any form (such as cigarettes, bidis, or gutkha)?

a. None ◯
b. 1 friend ◯
c. 2 friends ◯
d. 3 friends ◯
e. 4 or more friends ◯

Alcohol Use Behaviour

[These questions are on alcohol use behaviour. When we ask about drinking alcohol, we mean any alcoholic beverage, including beer, whiskey, wine, vodka, rum, scotch, fenni, breezers, cocktailsor locally manufactured alcohol]

41. Have you ever tried alcohol in any form?

a. Yes ◯
b. No ◯

41. (a). If yes, how old were you when you first tried alcohol?

__________ Years

42. During past 30 days, did you drink any type of alcohol?

a. Yes ◯
b. No ◯

43. How many of your friend/s drink any type of alcohol?

a. None ◯
b. 1 friend ◯
c. 2 friends ◯
d. 3 friends ◯
e. 4 or more friends ◯

D. INTENTIONS

Nutrition and physical activity

44. How often are you likely to do the following:

	Never	Sometime	Often	Very Often
a. Eat healthy food	◯	◯	◯	◯
b. Refuse /avoid junk food	◯	◯	◯	◯
c. Stay fit and exercise	◯	◯	◯	◯
d. Maintain healthy body weight for yourself	◯	◯	◯	◯

Tobacco

45. Do you think you will chew or smoke any type of tobacco when you enter college?

a. Surely Yes ◯
b. Maybe Yes ◯
c. Maybe No ◯
d. Surely No ◯

46. Do you think you will chew or smoke any type of tobacco when you are an adult?

a. Surely Yes ◯
b. Maybe Yes ◯
c. Maybe No ◯
d. Surely No ◯

47. If one of your friend offered a smoked/smokeless form of tobacco (cigarette, bidi, gutkha, khaini etc.) would you smoke/chew it?

a. Surely Yes ◯
b. Maybe Yes ◯
c. Maybe No ◯
d. Surely No ◯

48. If one of your family members offered a smoked/smokeless form of tobacco (cigarette, bidi, gutkha, khaini etc.) would you smoke/chew it?

a. Surely Yes ◯
b. Maybe Yes ◯
c. Maybe No ◯
d. Surely No ◯

Alcohol

49. Do you think you will try/start drinking alcohol when you enter college?

a. Surely Yes ◯
b. Maybe Yes ◯
c. Maybe No ◯
d. Surely No ◯

50. Do you think you will try/start drinking alcohol when you are an adult?

a. Surely Yes ◯
b. Maybe Yes ◯
c. Maybe No ◯
d. Surely No ◯

51. If one of your close friends offered an alcoholic drink; would you have it?

a. Surely Yes ◯
b. Maybe Yes ◯
c. Maybe No ◯
d. Surely No ◯

52. If one of your family members offered an alcoholic drink; would you have it?

a. Surely Yes ◯
b. Maybe Yes ◯
c. Maybe No ◯
d. Surely No ◯

E. PERCEPTIONS

Diet / Nutrition

53. How important are the following for you?

	Very Important	Important	Not Important	Not at all Important
a. Eating healthy food	○	○	○	○
b. Staying fit and exercising	○	○	○	○
c. Maintaining healthy weight	○	○	○	○
d. Looking attractive	○	○	○	○
e. Performing well in school	○	○	○	○
f. Doing well in sports	○	○	○	○
g. Eating at least one meal a day with family	○	○	○	○

54. How satisfied are you with your:

	Very Satisfied	Satisfied	Not Satisfied	Not at all Satisfied
a. Height	○	○	○	○
b. Weight	○	○	○	○
c. Waist size	○	○	○	○

55. Does any food or beverage advertisement seem attractive to you?

a. Yes ○
b. No ○

55. (a). If yes, please specify the brand of advertisement you like the most

a. Food ______________ b. Beverage ______________

Physical Activity

56. Do you think that increasing facilities in your school (sports equipment / improving school grounds etc.) will help you remain physically active?

a. Surely Yes ○
b. Maybe Yes ○
c. Maybe No ○
d. Surely No ○

57. Do you think that increasing Physical education/physical activity periods will help you remain physically active?

a. Surely Yes ○
b. Maybe Yes ○
c. Maybe No ○
d. Surely No ○

58. Do you think giving marks/grades for Physical education/physical activity subject may improve physical activity levels among students of your school?

a. Surely Yes ○
b. Maybe Yes ○
c. Maybe No ○
d. Surely No ○

Tobacco

59. How many boys of your age in your state do you think smoke tobacco regularly?

a. None (0%) ○
b. A few (1-33%) ○
c. Some (34-66%) ○
d. Most (67-100%) ○

60. How many boys of your age in your state do you think chew tobacco regularly?

a. None (0%) ○
b. A few (1-33%) ○
c. Some (34-66%) ○
d. Most (67-100%) ○

61. How many girls of your age in your state do you think smoke tobacco regularly?

a. None (0%) ○
b. A few (1-33%) ○
c. Some (34-66%) ○
d. Most (67-100%) ○

62. How many girls of your age in your state do you think chew tobacco regularly?

a. None (0%) ○
b. A few (1-33%) ○
c. Some (34-66%) ○
d. Most (67-100%) ○

63. How far do you agree with the following statements

	Strongly agree	Agree	Disagree	Strongly Disagree
a. It is fashionable to chew or smoke tobacco	○	○	○	○
b. It is fun to chew or smoke tobacco with friends	○	○	○	○
c. Using tobacco make a person appear to be more brave and grown up	○	○	○	○
d. Using tobacco reduce boredom (getting bored)	○	○	○	○
e. You would be more popular with your friends if you used tobacco	○	○	○	○

Alcohol

64. How many girls of your age in your state do you think drink alcohol regularly?

a. None (0%) ○
b. A few (1-33%) ○
c. Some (34-66%) ○
d. Most (67-100%) ○

10802110

65. How many boys of your age in your state do you think drink alcohol regularly?

- a. None (0%)
- b. A few (1-33%)
- c. Some (34-66%)
- d. Most (67-100%)

66. Why do you think under age youth / minors tend to drink alcohol?

	Surely yes	Maybe yes	Maybe no	Surely no
a. Parents alcohol intake	◯	◯	◯	◯
b. Friends/Peer alcohol intake	◯	◯	◯	◯
c. Peer Pressure	◯	◯	◯	◯
d. Easy availability/ affordability at Alcohol outlets, such as liquor stores, bars and restaurants	◯	◯	◯	◯
e. Advertising	◯	◯	◯	◯
f. Own decision	◯	◯	◯	◯
g. Lack of knowledge about harms of alcohol	◯	◯	◯	◯
h. Others, Please Specify ____				◯

F. FAMILY NORMS, PRACTICES & SUPPORT

67. How often do your family members do the following?

	Once in a while	Most of the time	All the time	Never
a. Limit your intake of sweets and sugary foods	◯	◯	◯	◯
b. Limit your intake of drinking soft drinks	◯	◯	◯	◯
c. Encourage you to eat healthy foods such as fruits, vegetables, whole grains	◯	◯	◯	◯
d. Eat meals with you	◯	◯	◯	◯
e. Physically active with you	◯	◯	◯	◯
f. Watch TV with you	◯	◯	◯	◯
g. Smoke at home	◯	◯	◯	◯
h. Chew tobacco at home	◯	◯	◯	◯
i. Consume alcohol at home	◯	◯	◯	◯

68. Do you have a TV or computer in your bedroom?

- a. Yes
- b. No

69. During the school days, do your parents limit how long you watch TV?

- a. No limits
- b. Yes, but not very strict limits
- c. Yes, strict limits
- d. I don't get much time for watching TV

70. During the school days, do your parents limit how long you use the computer (other than for doing homework/project work)?

- a. No limits
- b. Yes, but not very strict limits
- c. Yes, strict limits
- d. I don't get much time for using computer/ net surfing

71. Do any of your family members (staying with you in your house) smoke or chew tobacco in any form (such as cigarettes, bidis, or gutkha)?

- a. Yes
- b. No

72. Do any of your family members (staying with you in your house) drink any type of alcohol?

- a. Yes
- b. No

G. SCHOOL ENVIRONMENT

73. Which of the following are accessible to you during school hours at your school (Mark all that apply):

- a. Mid-day meal
- b. Canteen in school campus
- c. Food vendors/shops around school campus
- d. Playground
- e. Sport or exercise equipment
- f. Other Sports facilities (Badminton court, Tennis court, Swimming pool, running track in school campus)
- g. Yoga classes

74. In a typical week, how many Physical education/physical activity periods do you have in a week?

- a. None
- b. 1 per week
- c. 2-4 per week
- d. Everyday

75. Are students given marks / grades for physical education classes (including practical) in your school?

- a. Yes
- b. No

76. Is there is a canteen in your school ?

- a. Yes
- b. No

77. Do you know what items are sold in your school canteen

- a. There is no canteen in my school
- b. Yes
- c. No

77. (a) If yes, which of the following foods and drinks are available in your school canteens/cafeterias? (Mark all that apply)

Foods		Drinks	
a. Chocolates, sweets, candies	◯	a. Tea/coffee	◯
b. Pizza	◯	b. Packed juices (sweetened)	◯
c. Burger	◯	c. Packed juices (no added sugar)	◯
d. Samosa	◯	d. Carbonated drinks (sweetened eg. soft drinks)	◯
e. Puff/Pattie	◯	e. Carbonated drinks (unsweetened eg. soda, diet)	◯
f. Chips, wafers etc.	◯	f. Fruit beer	◯
g. Fried chaat	◯	g. Water	◯
h. French fries	◯	h. Milk / flavored milk	◯
i. Noodles	◯	i. Lassi / flavored lassi	◯
j. Salads	◯	j. Fresh fruit juice (no added sugar)	◯
k. Rajma rice	◯	k. Others (Please specify) ________	◯
l. Kadhi rice	◯		
m. Daal rice	◯		
n. Whole fruit	◯		
o. Others (Please specify) ________	◯		

78. Do you think the foods served in your school canteen are?

- a. There is no canteen in my school ◯
- b. Healthy ◯
- c. Unhealthy ◯
- d. Both healthy and unhealthy ◯
- e. Don't know ◯

79. Does your school canteen have restriction on the sale of any particular food items/drinks?

- a. There is no canteen in my school ◯
- b. Yes ◯
- c. No ◯
- d. Don't know ◯

80. Does your teacher/ assigned person/ class monitor check the food (lunchboxes) you brought from home?

- a. Yes ◯
- b. No ◯

80. (a) If yes, do they give/ write instructions for you / your parents to provide healthy food in lunch box ?

- a. Yes ◯
- b. No ◯

81. Does your school have any of the following display boards/ signage at the school entrance or in the school campus?

I. "Tobacco free school" board at a prominent place of the school entrance

- a. Yes ◯
- b. No ◯
- c. Don't know ◯

II. "No smoking area – smoking here is an offence" inside the school

- a. Yes ◯
- b. No ◯
- c. Don't know ◯

82. Have you ever observed sale of tobacco within 100 yards of your school campus?

- a. Yes ◯
- b. No ◯

83. Have you ever observed sale of alcohol within 75 meters in the vicinity of your school campus?

- a. Yes ◯
- b. No ◯

Thank you for filling this questionnaire.
Your help with this project is greatly appreciated.
